# HPLC Method for Determination of Enantiomeric Purity of a Novel Respiratory Fluoroquinolone: WCK 1152

**DOI:** 10.4103/0250-474X.43001

**Published:** 2008

**Authors:** R. D. Yeole, S. V. Lawand, S. B. Bhavsar, P. K. Deshpande

**Affiliations:** Wockhardt Research Center, D-4, MIDC, Chikalthana, Aurangabad-431 210, India

**Keywords:** Fluoroquinolone, enantiomer, WCK 1152, WCK 1153, enantiomeric purity, diastereomer

## Abstract

A sensitive, simple, specific, precise, accurate and rugged method for determination of enantiomeric purity of S-(-)-1-cyclopropyl-6-fluoro-1,4-dihydro-8-methoxy-7-{4-amino-3,3-dimethylpiperidin-1-yl}-4-oxo-quinoline-3-carboxylic acid hydrochloride monohydrate, WCK 1152, a new drug substance has been developed. The method is based on prederivatization of analyte to diastereomer followed by RP-HPLC using endcapped C-18 stationary phase. Column was maintained at 30°C. The UV/Vis detector was operated at 290 nm. Flow rate of the mobile phase was 1.25 ml/min. The method offers excellent separation of two enantiomers with resolution more than 4 and tailing factor less than 1.5. The method was validated for the quantification of R-(+)-enantiomer impurity, WCK 1153 in the bulk drug. Calibration curves showed excellent linearity over the concentration range of 0.1 to 1.5 mg/ml for WCK 1152 and 0.01 to 0.15 mg/ml for WCK 1153. Precision of the method was 1.13%. Limit of detection and limit of quantitation of the method for WCK 1152 were 0.0006 mg/ml and 0.0018 mg/ml and for WCK 1153 were 0.0007 mg/ml and 0.0021 mg/ml, respectively. Average recovery of the WCK 1153 in WCK 1152 was 94.4%. This method was employed in determining enantiomeric purity of clinical trial batches of WCK 1152.

During our consistent efforts to identify a novel broad-spectrum antibacterial agent we identified a potent fluoroquinolone, WCK 919. It was a mixture of two enantiomers, which were later denoted as WCK 1152 and WCK 1153. In order to differentiate activity and toxicity of these two isomers they were synthesized by using chiral resolution method. WCK 1152 and WCK 1153 were found to be very potent bactericidal. They were found to be highly potent against fluoroquinolone-resistant *Pneumococci, Viridans, Streptococci* and *Staphylococci*. These observations made them candidates for clinical development. WCK 1152 showed advantage over WCK 1153 in pre-clinical toxicity studies. Presently WCK 1152 is under clinical development for several indications. Chemically, WCK 1152 is an S-(-)-1-cyclopropyl-6-fluoro-1,4-dihydro-8-methoxy-7-{4-amino-3,3-dimethylpiperidin-1-yl}-4-oxo-quinoline-3-carboxylic acid hydrochloride monohydrate^1^–^5^. Its enantiomer WCK 1153 is chemically R-(+)-1-Cyclopropyl-6-fluoro-1,4-dihydro-8-methoxy-7-{4-amino-3,3-dimethylpiperidin-1-yl}-4-oxo-quinoline-3-carboxylic acid hydrochloride monohydrate. Chemical structures of WCK 1152 and WCK 1153 are shown in fig. 1. Due to the toxicity associated with WCK 1153, it was necessary to control and monitor its presence in WCK 1152. This article describes development and validation of a new analytical method, which can detect and quantify trace levels of WCK 1153 in WCK 1152.

**Fig. 1:**
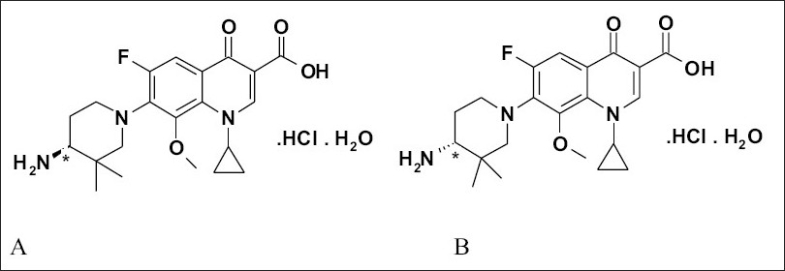
Chemical structures of WCK 1152 and WCK 1153. Chiral center is denoted by an asterisk in A) WCK 1152 and B) WCK 1153.

Several methods have been reported for determination of enantiomeric purity of fluoroquinolone antibacterial agents^6^. These methods include derivatization to diastereomers^7^–^10^, use of chiral mobile phase based on ligand exchange^11^–^13^ and application of chiral stationary phase (CSP) methods. Predominantly use of protein based CSPs and crown ether based CSPs have been reported^14^–^18^. In derivatization method enantiomers are reacted with chiral reagents to form a pair of diastereomers. These diastereomers posses different physical properties and hence can be separated on commonly used normal or reverse phase chromatography. In fluoroquinolones, generally two sites are available for derivatization, either carboxylic group of quinoline ring or amine group present in the side chain. Lehr *et al*^9^ have demonstrated separation of ofloxacin enantiomers after derivatization of carboxylic group with L-leucinamide. Similarly Machida *et al*^8^ have prepared diastereomers of gatifloxacin using L-valinamide and separated them on reverse phase chromatography. Foster *et al*^7^ derivatized secondary amino group of lomefloxacin with (S)-(+)-(1-napthyl)-ethyl isocyanate to diastereomers and separated them by normal phase chromatography. This article describes method based on derivatization of primary amine group present in side chain of WCK 1152 and WCK 1153 to diastereomers followed by reversed phase chromatography using C-18 column.

## MATERIALS AND METHODS

WCK 1152 and WCK 1153 were synthesized by Medicinal Chemistry group, Drug Discovery, Wockhardt Research Center. Both the compounds were characterized for their identity and purity. *Prima facie* their enantiomeric purity was monitored by specific rotation using polarimeter and were purified till equal opposite values for specific rotation were obtained. HPLC grade acetonitrile and methanol (Ranbaxy Fine Chemicals Limited, India), trifluoroacetic acid spectroscopy grade (E. Merck, Germany) and double distilled water passed through Purelab classic (US Filters) were used during studies.

HPLC system used was either from Shimadzu or Agilent. Shimadzu system comprised of degasser (DGU-14A), quaternary pump (LC-10ATvp), autoinjector (SIL-10ADvp), column oven (CTO-10ASvp) and UV/Vis detector (SPD-10Avp). The signal was acquired and processed using Class LC10 software. An Agilent-1100 series system comprised of degasser, quaternary pump, autoinjector, and column compartment and variable wavelength detector. The system was controlled through Chemstation software.

### Derivatization (preparation of diastereomers):

**Fig. 2:**
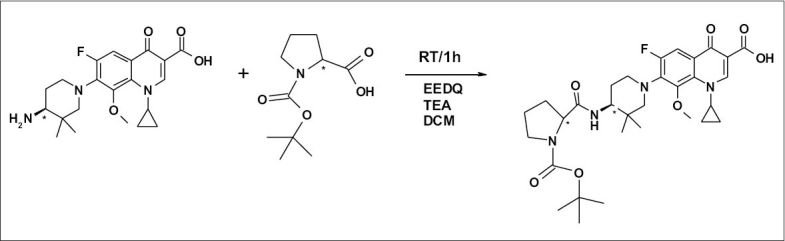
Reaction Scheme for Preparation of Derivative of WCK 1152. RT: Room temperature, EEDQ: N-ethoxycarbonyl-2-ethoxy-1,2-dihydroquinoline, h: hour, TEA: triethylamine, DCM: dichloromethane.

Derivatives of WCK 1152 and WCK 1153 were prepared by reacting them with N-boc-L-proline in presence of N-ethoxycarbonyl-2-ethoxy-1,2-dihydroquinoline (EEDQ) and triethylamine (TEA) in dichloromethane (DCM) as solvent at room temperature for 1 h. Completion of reaction was monitored by HPLC. Reaction scheme and chemical structure of the derivative is shown in fig. 2.

### Preparation of solutions and chromatographic conditions:

Solutions of N-boc-L-proline-WCK 1152 and N-boc-L-proline-WCK 1153 were prepared by dissolving weighed quantities in methanol. A solution of about 1 mg/ml was used for quantification purpose.

The chromatographic column used was a 250×4.6mm ID YMC Pack ODS AM (YMC Co. Ltd., Japan) with 5 μm particles. Mobile phase consist of a mixture of buffer and acetonitrile in proportion of 55.45. Buffer solution was prepared by dissolving 0.5 ml of trifluoroacetic acid in 1000 ml of water (pH of buffer 2.1). Flow rate of mobile phase was 1.25 ml/min. Column was maintained at 30°C and column eluent was monitored at 290 nm. Injection volume was 10 μl.

### System suitability:

Performance of the method was determined by injecting resolution mixture (1 mg/ml of N-Boc-proline derivative of WCK 1152 and 0.1 mg/ml of N-Boc-proline derivative WCK 1153) six times. Method performance criteria were resolution between two diastereomer peaks should be not less than 3.0, tailing factor not more than 1.5, and the precision (% RSD) of retention time and peak area of six repetitions should be less than 0.1 and 2.0%, respectively.

### Linearity:

Linearity of response for N-Boc-proline derivative of WCK 1152 and N-Boc-proline derivative of WCK 1153 was determined in the range of 0.1 to 1.5 mg/ml (10 to 150% of the assay solution concentration i.e. 1 mg/ml) and 0.01 to 0.150 mg/ml (10 to 150% of the specified limit i.e. 1% of WCK 1153 in WCK 1152), respectively.

### Limit of detection (LOD) and limit of quantification (LOQ):

LOD and LOQ of N-Boc-proline derivative of WCK 1152 and N-Boc-proline derivative of WCK 1153 were determined by calibration curve method^19^. Solutions of both the diastereomers were prepared in the range of 0.0005 to 0.05 mg/ml and injected in triplicate. Average peak area of three analyses was plotted against concentration. LOD and LOQ were calculated by using the equations, LOD= (Cd*Syx)/b and LOQ = (Cq*Syx)/b, where, Cd/Cq = coefficient for LOD/LOQ, Syx = residual variance due to regression and b = slope

### Precision and accuracy and ruggedness:

Precision of the method was determined by injecting six different preparations and determining % RSD of purity values. Accuracy of the method was determined by recovery studies. N-Boc-proline derivative of WCK 1153 was spiked in pre-analyzed sample of N-Boc-proline derivative of WCK 1152 and its percent recovery was determined. Ruggedness of the method was determined by performing quantification of N-Boc-proline derivative of WCK 1153 on two different HPLC systems and columns by two analysts.

## RESULTS AND DISCUSSION

The objective of this work was to develop a precise and accurate method to determine enantiomeric purity of WCK 1152 preferably without using expensive chiral stationary phases. Various options were attempted to develop such method. In first attempt chiral mobile phase additives were used e.g. L-phenylalanine, beta-cyclodextrin and L-tryptophan. Use of beta-cyclodextrin and L-tryptophan could not provide any separation of enantiomers. Little success was achieved with use of L-phenyl alanine. Resolution of 1.3 was obtained by using L-phenylalanine at pH 4 using base deactivated octadecyl silane as stationary phase. This separation was insufficient to quantify presence of less than 1% unwanted enantiomer. Another methodology attempted was derivatization of analyte to diastereomer and then separate them chromatographically. Since chiral center is present in the side chain of WCK 1152 attempts were made to derivatize primary amine group. First derivative prepared was with Mosher acid. Excellent resolution of 4.3 was obtained on silica gel column using isopropyl alcohol and hexane as mobile phase. To lessen the cost of analysis another derivative was prepared using protected L-proline. The diastereomers were separated on reverse phase chromatography with resolution more than 4 and tailing factor of less than 1.5. This method was further validated for estimation of WCK 1153 (unwanted enantiomer) in WCK 1152 bulk drug. As mentioned above application of chiral mobile phase additive did not give acceptable resolution required to quantify presence of ≤1% of WCK 1153 in WCK 1152. Two enantiomers were well separated after derivatization with protected L-proline.

**Fig. 3:**
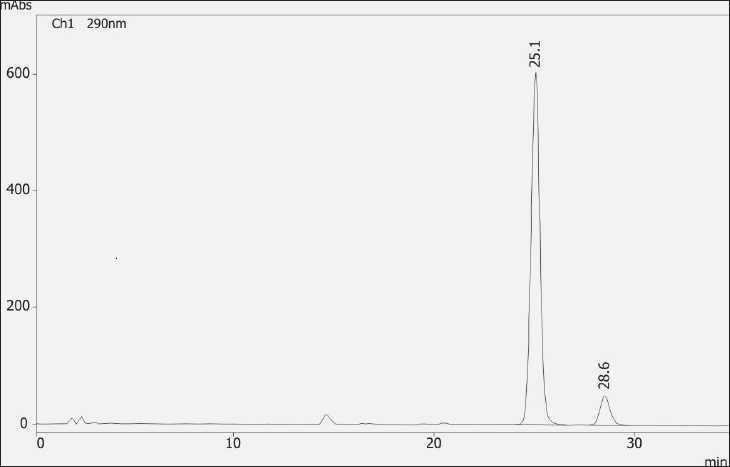
HPLC Chromatogram of WCK 1152 and WCK 1153. Chromatograms with peak of N-Boc-proline derivative of WCK 1152 (RT = 25.1 min) and N-Boc-proline derivative of WCK 1153 (RT = 28.6 min)

A representative chromatogram showing resolution of derivatives of enantiomers is shown in fig. 3. An excellent resolution (Rs = 4.08) between the two peaks along with tailing factor for N-Boc-proline derivative of WCK 1152 and N-Boc-proline derivative of WCK 1153 was 1.04 and 1.17, respectively, were obtained. Numbers of theoretical plates were more than 15000 for both the diasteromers. Precision (RSD) of injection repeatability was found to be 1.46% and 1.51% for pair of diasteromers. The described method was found to be linear for N-Boc-proline derivative of WCK 1152 in the range of 0.1 to 1.5 mg/ml (10 to 150% of the assay solution concentration i.e. 1 mg/ml). Method was also found to be linear for N-Boc-proline derivative of WCK 1153 in the range of 0.01 to 0.150 mg/ml (10 to 150% of the specified limit i.e. 1% of WCK 1153 in WCK 1152). Correlation coefficients obtained were 0.9971 and 0.9986 for N-Boc-proline derivative of WCK 1152 and N-Boc-proline derivative of WCK 1153, respectively. LOD and LOQ for both the enantiomers were determined by calibration curve method. LOD and LOQ for N-Boc-proline derivative of WCK 1152 were found to be 0.0006 mg/ml and 0.0018 mg/ml, respectively. LOD and LOQ for N-Boc-proline derivative of WCK 1153 were found to be 0.0007 mg/ml and 0.0021 mg/ml, respectively. Calculations were performed by considering values 3.3 and 10 for Cd and Cq, respectively. Mean recovery of N-Boc-proline derivative of WCK 1153 was found to be 94.4%. Amount spiked and amount found of N-Boc-proline derivative of WCK 1153 are shown in Table 1. The method was found to be rugged as content of N-Boc-proline derivative of WCK 1152 and N-Boc-proline derivative of WCK 1153 did not deviate significantly on two systems with overall relative standard deviation 0.003 and 1.644%, respectively. Ruggedness data for N-Boc-proline derivative of WCK 1152 is shown in Table 2 and ruggedness data for N-Boc-proline derivative of WCK 1153 is shown in Table 3.

**TABLE 1:** RECOVERY OF N-BOC-PROLINE DERIVATIVE OF WCK 1153

Amount added (%)	Amount found (%)	Recovery (%)	Mean	SD	RSD (%)
Nil	0.1713				
0.8	0.8893	89.75	89.17	0.51	0.58
	0.8831	88.98			
	0.8815	88.78			
1	1.1789	100.76	100.75	0.17	0.16
	1.1771	100.56			
	1.1804	100.91			
1.2	1.2957	93.70	93.23	0.58	0.62
	1.2923	93.42			
	1.2823	92.58			

SD: Standard deviation, RSD: Relative standard Deviation

**TABLE 2:** RUGGEDNESS DATA OF N-BOC-PROLINE DERIVATIVE OF WCK 1152

Data	N-Boc-L-proline WCK 1152 content (%)		
	
	System-1		System-2
1	99.83		99.83
2	99.83		99.83
3	99.82		99.83
4	99.83		99.83
5	99.82		99.83
Mean	99.826		99.83
SD	0.005		0
RSD (%)	0.005		0
Overall mean		99.828	
SD		0.003	
RSD (%)		0.003	

SD: Standard deviation, RSD: Relative standard deviation

**TABLE 3:** RUGGEDNESS DATA OF N-BOC-PROLINE DERIVATIVE OF WCK 1153

Data	N-Boc-L-proline WCK 1153 content (%)		
	
	System-1		System-2
1	0.17		0.17
2	0.17		0.17
3	0.18		0.17
4	0.17		0.17
5	0.18		0.17
Mean	0.174		0.17
SD	0.005		0
RSD (%)	3.148		0
Overall mean		0.172	
SD		0.003	
RSD (%)		1.644	

SD: Standard deviation, RSD: Relative standard deviation

A simple, precise, accurate and rugged reverse phase HPLC method based on derivatization of enantiomers has been developed and validated for quantitative determination of unwanted enantiomer WCK 1153 in new drug substance WCK 1152. This method was used for monitoring enantiomeric purity of WCK 1152 clinical trial batches. Method was also used for determining any racemization of WCK 1152 to WCK 1153 in clinical trial samples i.e. serum and urine with an additional step of extraction of analyte from biological matrices using solid phase extraction.
